# Trends of Postpartum Depression in Iran: A Systematic Review and Meta-Analysis

**DOI:** 10.1155/2013/291029

**Published:** 2013-07-09

**Authors:** Yousef Veisani, Ali Delpisheh, Kourosh Sayehmiri, Shahab Rezaeian

**Affiliations:** ^1^Student Research Committee, Ilam University of Medical Sciences, P.O. Box 69311-57793, Ilam, Iran; ^2^Department of Clinical Epidemiology, Faculty of Medicine, Ilam University of Medical Sciences, P.O. Box 69315-138, Ilam, Iran; ^3^Prevention of Psychosocial Injuries Research Centre, P.O. Box 69311-57793, Ilam, Iran; ^4^Department of Epidemiology, School of Health & Nutrition, Shiraz University of Medical Sciences, P.O. Box 71348-14366, Shiraz, Iran

## Abstract

*Background*. Postpartum depression (PPD) is a serious mental health disorder affecting 13% of women in developed communities. The present study reviews available epidemiological publications on PPD-related aspects in Iranian women to help policy makers and health workers to design preventative strategies and further researches. *Materials and Methods*. A systematic review was constructed based on the computerized literature valid database. The 95% confidence intervals were calculated by random effects models. Metaregression was introduced to explore and explain heterogeneity between studies. Data manipulation and statistical analyses were performed using Stata 11. *Results*. Overall, 41 studies met the inclusion criteria. The pooled prevalence of PPD in Iran was 25.3% (95% CI: 22.7%–27.9%). Amongst subgroups of unwanted delivery, illiterate, housewives, and having history of depression the prevalence was 43.4% (35.6–51.1), 31.6% (18.1–45.0), 30.7% (25.2–36.3), and 45.2% (35.4–53.1), respectively. *Conclusions*. Interventions that would specifically target women with a prior history of depression, illiterates, housewives, or women with unwanted pregnancies could be helpful to decrease the prevalence of postpartum depression in Iran.

## 1. Introduction

Postpartum depression (PPD) affects almost 13% of women in developed high income communities [1] and may be even more common in developing countries [2, 3]. According to the Diagnostic and Statistical Manual of Mental Disorders, Fourth Edition (DSM-IV), PPD is a major depression when symptoms have onset within 5 weeks of childbirth [4]. PPD presents with the same symptoms as for a major depressive episode occurring outside of the prenatal period, including core symptoms of depressed mood and/or loss of pleasure, together with additional symptoms, including changes in weight or sleep, fatigue or loss of energy, feelings of worthlessness or guilt, concentration difficulties, and suicidal ideation [4].

Majority of PPD researches in Iran have not utilized diagnostic assessments to identify cases. Alternatively, they have used the validated self-report depression screening instruments, such as the Edinburgh Postnatal Depression Scale (EPDS) [5]. Although this approach has been criticized, the EPDS has showed good sensitivity and specificity, particularly when used to detect both major and minor depressions [5].

In terms of etiology, PPD is a multifactorial disorder with biological, psychological, and sociological aspects interacting with woman's risk individually [6]. Sociological factors such as unwanted delivery, occupation, literacy, and history of depression have been more frequently reported throughout original researches and a meta-analysis [7]. However, they are not comprehensive as the present review is. Many of them drew incompatible or even contradictory conclusions, and the utilization of these statistics is therefore limited. The present study reviews available epidemiological publications on PPD-related aspects in Iranian women to help policy makers and health workers to design preventative strategies and further researches.

## 2. Methods and Materials

### 2.1. Literature Search

Our search strategy, selection of publications, and the reporting of results for the review will be conducted in accordance with the PRISMA guidelines [8]. Literature on the postpartum depression among Iranian women was acquired through searching the Scientific Information Databases (SID), Global Medical Article Limberly (Medlib), Iranian Biomedical Journal (Iran Medex), and Iranian Journal Database (Magiran) as well as international databases including PubMed/Medline, Scopus, and ISI Web of Knowledge. The search strategy was limited to the Persian and/or English language papers published until Feb 2012. All publications with medical subject headings (MeSh) and keywords in title, abstract, and text for words including postpartum depression were investigated. Iranian scientific databases were searched only using the keyword “postpartum depression,” as these databases do not distinguish synonyms from each other and do not allow sensitive search operation using linking terms such as “AND,” “OR,” or “NOT.” Consequently, this single keyword search was the most practical option. The postpartum depression, depression, and Iran MeSh combined with the Selection and Quality Assessment of Articles operator “OR” versus “AND.”

### 2.2. Selection and Quality Assessment of Articles

All identified papers were critically appraised independently by two reviewers. Disagreements between reviewers were resolved by consensus. Appraisal was guided by a checklist assessing clarity of aims and research questions. The inclusion criteria were as follows: (1) studies in the mentioned databases with full text, despite the language of original text; (2) having a standardized assessment of depression (either self-report or observer rated), using Edinburgh Postnatal Depression Scale (EPDS) and Beck depression inventory (BDI) instruments and study conducting of between 2 and 52 weeks postpartum were the main inclusion criteria. Exclusion criteria were (1) studies upon women overlapping time intervals of sample collection from the same origin; (2) inappropriate study design; (3) inadequate reporting of results.

### 2.3. Data Extraction

Data were extracted using a standardized and prepiloted data extraction form. Data extraction will be undertaken by the first reviewer and checked by a second reviewer although the process will be discussed and piloted by both reviewers. All identified papers will be critically appraised independently by both reviewers. Disagreements were resolved through discussion. Appraisal will be guided by a checklist assessing clarity of aims and research questions. Information was extracted from each included study (including author, title, year and setting of study, methods of sample selection, sample size, study type, age, and prevalence). Therefore, risk of bias as an “Inadequate Reporting” reduced. These data-abstraction forms were reviewed, and eligible papers were entered into the meta-analysis. Besides, as with all meta-analyses, this study has potential limitation of publication bias. Many of our data were extracted from studies written in Persian (language bias). However, we have confidence in our results since the included literature was published in non-Persian language, which should reduce publication bias to some extent.

### 2.4. Statistical Analysis

The random effects model was used for combining results of studies in meta-analysis. Variance for each study was calculated using the binomial distribution formula. The presence of heterogeneity was determined by the Der Simonian-Laird (DL) approach [9]. Significance level was <0.1, and *I* ^2^ statistic was used for estimates of inconsistency within the meta-analyses. The *I* ^2^ statistic estimates the percent of observed between-study variability due to heterogeneity rather than to chance and ranges from 0 to 100 percent (values of 25%, 50%, and 75% were considered representing low, medium, and high heterogeneity resp.) [10]. A value of 0% indicates no observed heterogeneity whilst 100% indicates significant heterogeneity. For this review, we determined that *I* ^2^ values above 75 percent were indicative of significant heterogeneity warranting analysis with a random effect model as opposed to the fixed effects model to adjust for the observed variability. The *I* ^2^ is an estimate of the proportion of the total variation across studies that are beyond chance. In situations with high between-study heterogeneity, the use of random effects models is recommended as it produces study weights that primarily reflect the between-study variation and thus provide close to equal weighting. Univariate and multivariate metaregression analyses were used to explore possible sources of heterogeneity among studies [11]. We analyzed sources of heterogeneity by subgroup and metaregression analysis using dichotomous and continuous variables. Univariate and multivariate approaches were employed to assess the causes of heterogeneity among the selected studies. Metaregression was used to show the trend of variation of prevalence during time. Egger test was conducted to examine potential publication bias. Egger's test can reveal a symmetric or asymmetric funnel plot. The latter indicates the existence of a significant publication bias or a systematic heterogeneity between studies. Data manipulation and statistical analyses were done using STATA software, version 11.2. *P* values <0.05 were considered as statistically significant.

## 3. Results

**Figure 1:**
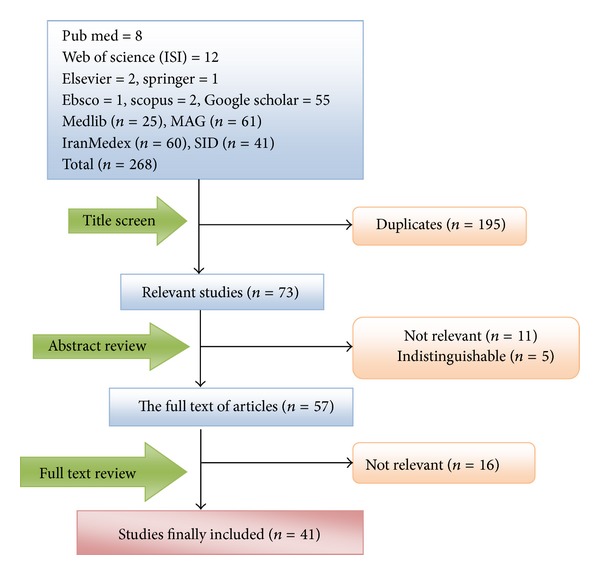
Results of the systematic literature search.

According to the literature search strategies, 268 studies were identified, but 227 studies were excluded as they did not meet the inclusion criteria. There were 9 studies in English [5, 12–19] and 32 studies in Persian [20–51] of the finally adopted 41 studies, and they were published between 1995 and 2012. The pooled sample sizes included 21907 women (Table 1 and Figure 1).

**Figure 2:**
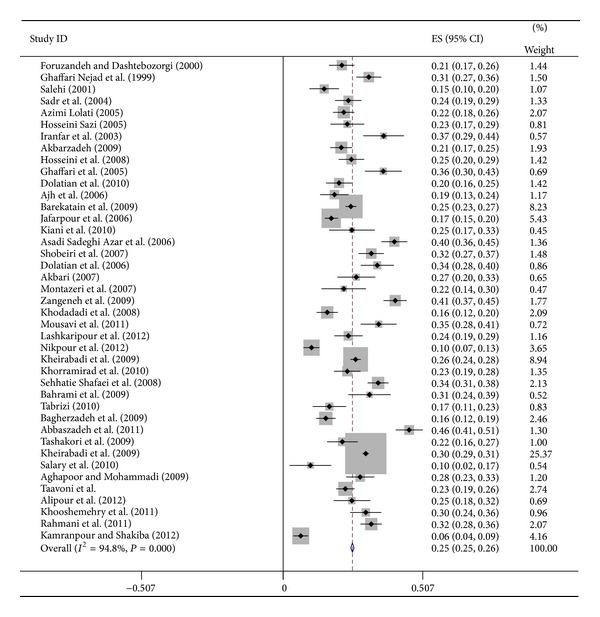
Forest plots for random effects meta-analyses. CI indicates confidence interval.

The heterogeneity between studies was 94.5% with an *I* square (*I* ^2^) statistic (*P* ≤ 0.001). The pooled prevalence of postpartum depression was 25% (95% CI: 22.7–27.9%) (Figure 2). Based on the Edinburgh Postnatal Depression Scale (EPDS) and Beck depression inventory (BDI), the PPD prevalence in Iran was 24.3% (95% CI: 21.0–27.7) and 25.3% (95% CI: 22.7–27.9), respectively.

Amongst subgroups of unwanted delivery, illiterate, housewives, and having history of depression, the prevalence was 43.4% (35.6–51.1), 31.6% (18.1–45.0), 30.7% (25.2–36.3), and 45.2% (35.4–53.1), respectively (Table 2). A significant geographic difference in pooled PPD was observed.

**Figure 3:**
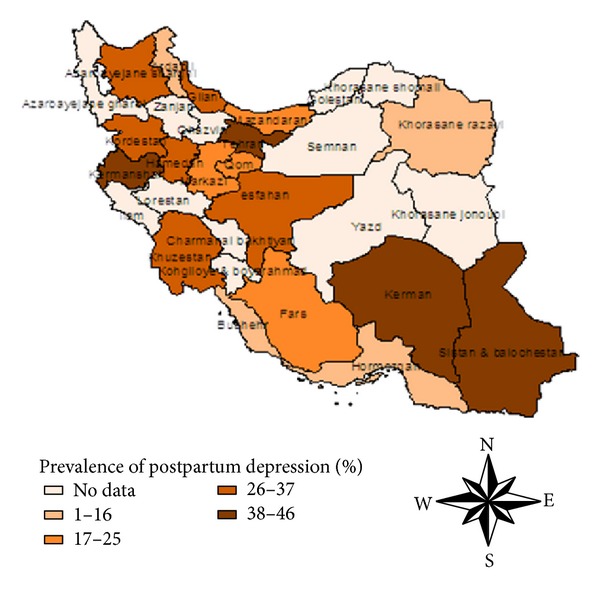
Regional distribution of pooled prevalence of postpartum depression in Iran.

The lowest PPD rate was observed in central, and the highest rate was observed in west and south-eastern border areas of Iran (Figure 3). 

**Figure 4:**
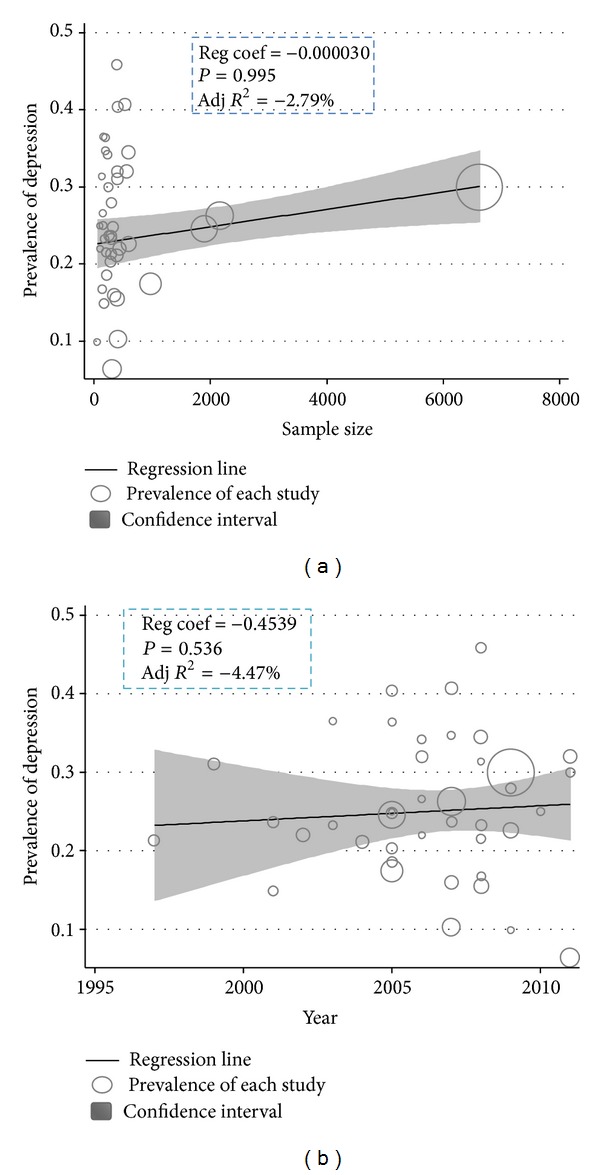
Metaregression plots of change in PPD according to changes in continuous study moderator's year and sample size.

**Figure 5:**
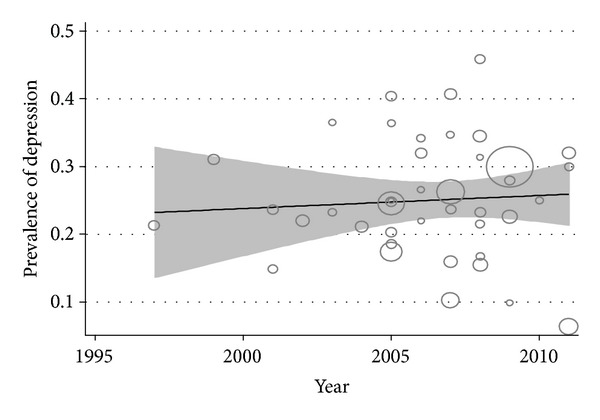
Prevalence of postpartum depression among Iranian women at different study periods.

The metaregression of the prevalence PPD for each study on the interval sample size showed a negative and no statistically significant relationship (*β* = −0.0003, s.e. (*β*) = 0.0002, *P* = 0.995) and no statistically significant change in prevalence over the time (*β* = −0.4539, s.e. (*β*) = 0.025, *P* = 0.536) (Figure 4). Since 1995, the PPD rates showed an increasing trend (Figure 5).

## 4. Discussion

Comprehensiveness of available information and a large sample size made the present study representative. There have been recent systematic reviews of studies dealing with risk factors of PPD in Iranian women [7], but the present study aimed to determine prevalence of PPD by a systematic review and meta-analysis method. The psychometric properties of two main screening tools such as the Edinburgh Postnatal Depression Scale (EPDS) and Beck depression inventory (BDI) have been repeatedly assessed across the world.

The present study showed that the pooled prevalence of PPD among Iranian women was 25.3%, which is similar to the finding of a recent meta-analysis reported by Paulson and colleagues (25.6%) [54]. But it was not consistent with another meta-analysis of 15.6% [55]. This difference may be due to the assessment tools, geographic, and cut-off point differences. Prior studies that have noted the importance of recurrent PPD in women with a prior episode of postpartum affective psychosis may be at risk for recurrence postpartum 50–70% [56].

A significant geographic difference of the prevalence of PPD was also observed. Compared with other regions, west and south-eastern borders of Iran had relatively higher prevalence rates, accounting for 48.7% and 40.4%, respectively. Meanwhile, the lowest prevalence rate was found in north of Iran of 6.4%. Notably, during the period through 1995 and 2012, the prevalence rates were commonly at high level among different regions of Iran especially in border regions; the rates even reached nearly 50%. Possible reasons for this lack of reduction may include backwardness of border province to the central provinces, poor economic conditions, lack of program education in the sensitive groups, or limited sampling.

An extensive list of characteristics of PPD was examined among a diverse and representative sample of Iranian women. Conducting this study with a large sample size increased the statistical power. Sociological factors in Iran such as unwanted delivery as a result of the lack of family planning, illiterate result of gender discrimination, and carrier because most women are housewives have been more studied. The results of this study indicate that prevalence of PPD in illiterate women, unwanted delivery, and housewives women with a prior history of depression was 31.6%, 43.4%, 30.7%, 45.2%, respectively. Rates of relapse are particularly high in women with a prior history of depression with estimates ranging from 25%–50% [57]. Interventions that would specifically target women with a prior history of depression, illiterate women, housewives mothers, or women with unwanted pregnancy may help to decrease the prevalence of PPD among this population.

The strengths of this review include the large number of samples included and therefore the ability to examine prevalence in clinically relevant subgroups with some degree of precision. The *I* ^2^ is an estimate of the proportion of the total variation across studies that is beyond chance. In situations with high between-study heterogeneity, the use of random effects models is recommended as it produces study weights that primarily reflect the between-study variation and thus provides close to equal weighting. Univariate and multivariate metaregression analyses were used to explore possible sources of heterogeneity among studies [11]. Furthermore, we have examined heterogeneity using subgroup analyses and metaregression, which allowed us to investigate dichotomous and continuous variables such as age, sample size, and the date when the study was conducted. According to the metaregression analysis, none of the data were not statistically significant with changes of PPD prevalence. The high levels of heterogeneity between the studies are to be expected as the studies were conducted in different samples, and this may simply reflect real differences in prevalence over time and by region. Particular reasons for this difference in women are unclear. Our approach to this was to identify causes of heterogeneity, and two possible explanations were assessed. Possibilities include that in the diagnostic systems, fatigability is included in the core criteria for depression. In addition, it may be that the distinction between minor and major forms of depression is more important in women as the overlap between sadness and clinical depression is more difficult to determine.

There are some limitations in the present study which need to be addressed. First of all, more studies were observational and patients were not randomly chosen. Therefore, selection bias and confounding seem inevitable. Secondly, much of our data were extracted from the internal databases in Iran. Thirdly, our ability to assess study quality was limited by the fact that many studies failed to offer detailed information on selected subjects or valid data on important factors, and in the end timing of the administration of the EPDS and information bias may be present due to the self-report nature, and eventually in this review, there may be other explanations for the heterogeneity that we did not test, such as comorbidity with other mental disorders, but systematic data on this were lacking.

## Figures and Tables

**Table 1:** Feature of studies among women with postpartum depression at different regions.

Study number/author(s)	Place	No. of population	Assessment times	Instrument Assessment	Cut point	Prevalence of PPD (%)
(1) Kheirabadi et al. [12, 16]	Isfahan	6627	2−12 weeks	EPDS	13	30
(2) Khorramirad et al. [38]	Qom	300	6−12 weeks	EPDS	13	23.7
(3) Hosseini Sazi et al. [34]	Gorgan	180	2−14 weeks	BDI	16	23.3
(4) Zangeneh et al. [51]	Kermanshah	531	2−6 weeks	EPDS	13	40.7
(5) Azimi Lolati et al. [24]	Sari	442	6−8 weeks	EPDS	13	22
(6) Sehhatie Shafaei et al. [49]	Tabriz	600	10−20 weeks	EPDS	12	34.7
(7) Shobeiri et al. [50]	Hamadan	400	2−8 weeks	BDI	16	32
(8) Khodadadi et al. [36]	Rasht	350	6−8 weeks	EPDS	13	16
(9) Hosseini et al. [33]	Kermanshah	330	4−14 weeks	BDI	16	24.8
(10) Ghaffari et al. [31]	Ramsar	100	6−16 weeks	GHQ 28	22	36.6
(11) Foruzandeh and Dashtebozorgi [30]	Shahrekord	300	6−10 weeks	BDI	16	21.3
(12) Bahrami et al. [26]	Dezful	140	6−10 weeks	EPDS	13	31.4
(13) Dolatian et al. [28]	Tehran	285	2−6 weeks	EPDS	12	20.3
(14) Ajh et al. [22]	Astaneh	440	2−4 weeks	BDI	16	18.6
(15) Khooshemehry et al. [37]	Tehran	250	6−52 weeks	BDI	16	30
(16) Tabrizi et al. [52]	Hamadan	144	2−12 weeks	BDI	16	16.8
(17) Barekatain et al. [27]	Isfahan	1898	6−8 weeks	EPDS	13	24.4
(18) Rahmani et al. [45]	Tabriz	560	8−52 weeks	EPDS	12	32
(19) Jafarpour et al. [35]	Kermanshah	975	13−26 weeks	EPDS	12	17.5
(20) Mousavi et al. [42]	Kashan	204	9−13 weeks	BDI	13	34.7
(21) Bagherzadeh et al. [25]	Bushehr	400	2−12 weeks	EPDS	15	15.5
(22) Kiani et al. [39]	Astara	105	2−4 weeks	EPDS	12	25
(23) Kamranpour and Shakiba [20]	Rasht	310	2−8 weeks	EPDS	12	6.4
(24) Dolatian et al. [28, 29]	Marivan	204	2−6 weeks	EPDS	10	34.2
(25) Salary et al. [47]	Mashhad	60	2−4 weeks	EPDS	10	9.9
(26) Lashkaripour et al. [40]	Zahedan	300	4−18 weeks	BDI	15	33.7
(27) Nikpour et al. [43]	Amol	420	2−8 weeks	EPDS	12	10.3
(28) Asadi Sadeghi Azar et al. [13]	Zabol	408	2−8 weeks	BDI	16	40.4
(29) Aghapoor and Mohammadi [21]	Tabriz	300	6−12 weeks	BDI	16	28
(30) Ghaffari Nejad et al. [32]	Kerman	400	2−8 weeks	BDI	16	31.1
(31) Akbari et al. [53]	Hamadan	159	4−8 weeks	EPDS	13	26.4
(32) Salehi [48]	Hormozgan	164	2−8 weeks	EPDS	13	14.9
(33) Sadr et al. [46]	Tehran	300	2−8 weeks	EPDS	13	23.7
(34) Taavoni et al. [14]	Tehran	597	4−12 weeks	EPDS	13	22.6
(35) Alipour et al. [15]	Qom	160	4−12 weeks	EPDS	13	25
(36) Montazeri et al. [5]	Isfahan	100	12−14 weeks	EPDS	13	22
(37) Kheirabadi et al. [12, 16]	Isfahan	1291	6−8 weeks	EPDS	13	26.3
(38) Iranfar et al. [17]	Kermanshah	163	2−8 weeks	BDI	10	48.7
(39) Abbaszadeh et al. [18]	Kerman	400	8−28 weeks	EPDS	13	45.8
(40) Tashakori et al. [19]	Ahvaz	210	8−28 weeks	EPDS	12	21.4
(41) Akbarzadeh et al. [23]	Shiraz	400	2−8 weeks	BDI	16	21.1

**Table 2:** Prevalence of postpartum depression among subgroups.

Variables	No. of studies	No. of patients	Prevalence % (95% CI)	Heterogeneity		Model
				*I* ^2^	*P* value	
Housewife	8	10756	30.7 (25.2–36.3)	96.4%	0.000	REM*
Employee	8	10756	29.3 (21.8–36.8)	96.4%	0.000	REM
History of depression	8	10787	45.2 (35.4–53.1)	97.9 %	0.000	REM
No history of depression	8	10787	27.3 (21.1–33.5)	97.3%	0.000	REM
Unwanted delivery	11	11702	43.4 (35.6–51.1)	98.3%	0.000	REM
Desired delivery	11	11702	29.0 (23.5–34.6)	97.1%	0.000	REM
Illiterate	4	7688	31.6 (18.1–45.0)	98.3%	0.000	REM
literate	4	7688	41.1 (25.1–57.1)	98.7%	0.000	REM
Pooled	41	21907	25.3 (22.7–27.9)	94.8%	0.000	REM

*Random effects model.
